# Disposition of patients with coronavirus disease 2019 (COVID-19) whose respiratory specimens remain positive for severe acute respiratory coronavirus virus 2 (SARS-CoV-2) by polymerase chain reaction assay (PCR)

**DOI:** 10.1017/ice.2020.286

**Published:** 2020-06-10

**Authors:** Leonard A. Mermel

**Affiliations:** 1 Division of Infectious Diseases, Department of Medicine, Warren Alpert Medical School of Brown University, Providence, Rhode Island; 2 Department of Epidemiology and Infection Control, Rhode Island Hospital, Providence, Rhode Island

Patients with coronavirus disease 2019 (COVID-19) infection may respiratory tract specimens positive for severe acute respiratory coronavirus virus 2 (SARS-CoV-2) for several days while in isolation precautions or for several weeks after removal from isolation precautions. This situation creates a dilemma for infection control staff in acute and chronic healthcare settings and for public health officials. Potentially keeping such patients in isolation precautions beyond the period when they are no longer infectious leads to unnecessary actions and consequences: personal protective equipment use; single-patient room occupancy; repeat PCR testing; social isolation; delay from reentering the work force, particularly problematic for healthcare workers and essential civil servants; and redistribution of infection control and public health human capital assigned to track the status of such individuals. These downstream effects have unequivocal and profound economic impact that may be avoidable.

## Can patients in isolation precautions with COVID-19 infection be removed from precautions prior to current guidelines?

Yes isolation precautions can be removed after 9 days from symptom onset or after 9 days from the first positive SARS-CoV-2 PCR test of a respiratory specimen in asymptomatic individuals.

No reports, known to this author, of viable SARS-CoV-2 detection in respiratory tract specimens collected beyond 9 days after symptom onset have been published.^1-3^ As with other emerging viral infections,^4^ patients with a high SARS-CoV-2 PCR cycle threshold (eg, cycle threshold ≥34 in one study^5^ or >24 in another^3^) have not been found to have live virus in their respiratory secretions. Similar findings at CDC have been found with a cycle threshold >33 with the N1 amplification target.^6^

A study in Taiwan^7^ included 100 patients with COVID-19 infection and their 2,761 close contacts (face-to-face contact for >15 minutes with a confirmed COVID-19 patient). SARS-CoV-2 PCR testing was performed on all symptomatic contacts (ie, contacts with fever, cough, or other respiratory symptoms). SARS-CoV-2 polymerase chain reaction (PCR) testing was also performed on all household and hospital contacts, regardless of symptoms, when they were initially assessed. If PCR testing was negative, they were tested again if they developed such symptoms during the 14 days after their initial contact with a case patient. The investigators found no secondary COVID-19 infections among 852 contacts exposed to infected cases if the exposure occurred after the initial 5 days of symptom onset. These epidemiologic data support the aforementioned laboratory data.

## Are there exceptions?

Yes, severely immunocompromised patients may be an exception. Based on data from other viral infections,^8,9^ patients with COVID-19 infection who are severely immunocompromised may have prolonged shedding of live virus. Thus, decisions regarding discontinuing isolation precautions for severely immunocompromised patients, or possibly those who are otherwise critically ill with COVID-19 infection, should be based on a high SARS-CoV-2 PCR cycle threshold.^3,5,6^ Importantly, the cycle threshold varies depending on the PCR protocol and amplification target used in the PCR assay. Such patients, and all others, should continue to follow CDC and local health official guidance regarding continued source control after hospital discharge (ie, mask use and hand hygiene), as well as social distancing.

## Are patients infectious if they previously had a COVID-19 infection, met criteria for removal from isolation precautions, and they have SARS-CoV-2 PCR-positive respiratory tract specimens over the next several weeks?

In most cases, no. The Korean CDC studied 285 SARS-CoV-2 PCR-positive patients after removal from isolation precautions and an average of 45 days after symptom onset (range, 8–82 days); 126 still had some COVID-19 related symptoms.^10^ All 285 were seropositive. SARS-CoV-2 culture was negative in 108 patients who had such cultures performed. These 285 patients had 790 contacts, including 351 family members. Contacts were monitored for minimum of 14 days each. SARS-CoV-2 PCR testing of contacts was performed if they became symptomatic (ie, either temperature ≥37.5°C, sore throat, cough, etc); otherwise, PCR testing was done on day 13 after the exposure if the contact was a healthcare worker or household member (YJ Choe, personal communication). There was no evidence of COVID-19 transmission to these contacts: 27 of the 790 contacts were previously SARS-CoV-2 PCR-positive, and 3 newly SARS-CoV-2 PCR-positive contacts had other high-risk exposures.

## Are there exceptions?

Yes, severely immunocompromised patients or reinfection in those patients or others may be exceptions. For severely immunocompromised patients or if otherwise in question, the SARS-CoV-2 cycle threshold will assist in determining infectivity. Reinfection with SARS-CoV-2 remains an open question. At this time, it is unclear when to assess patients for possible reinfection and the risk of disease transmission if that occurs. After COVID-19 infection, SARS-CoV-2 IgG antibodies remain significantly elevated for at least 7 weeks in most cases^11^; however, 6% of patients with relatively mild COVID-19 infection have been found to recover without detectable neutralizing antibodies.^12^ Neutralizing antibodies can be detected for 2 years in ~90% SARS-infected patients.^13^ However, antibody levels drop after 2–3 years in patients who recovered from SARS and MERS-CoV infections.^14^ Reinfection from the same genotype of human coronaviruses can occur within months to a year later.^15,16^ Since SARS-CoV-2 neutralizing antibodies are protective in rhesus macaques,^17,18^ and if durability of these antibodies is similar to that of patients who recovered from SARS and MERS-CoV infections, SARS-CoV-2 PCR positivity beyond 9 days from symptom onset is unlikely to reflect reinfection over the ensuing months in seropositive immunocompetent patients. However, if neutralizing antibody levels wane after several months to a year, then SARS-CoV-2 PCR positivity may reflect reinfection, and the SARS-CoV-2 cycle threshold will assist in determining the need for isolation precautions or quarantine.

Based on the aforementioned data, patients with COVID-19 infection who are beyond 9 days from symptom onset or beyond 9 days from the first SARS-CoV-2 PCR-positive testing of a respiratory specimen in asymptomatic patients, should not undergo repeat SARS-CoV-2 PCR testing unless they are presenting several months after symptom onset or asymptomatic detection (ie, long enough time for possible reinfection), or they are otherwise severely immunocompromised. The patient should not be placed back in isolation precautions unless severely immunocompromised. Immunocompetent patients with a SARS-CoV-2 PCR-positive respiratory specimen obtained >9 days after symptom onset, or first positive testing for asymptomatic patients, should be allowed to have procedures, surgical or otherwise, or to undergo testing as clinically indicated without the precautions used for patients with active COVID-19 infection unless they are presenting several months after either symptom onset or their initial positive SARS-CoV-2 PCR testing, or they are severely immunocompromised. In such cases, determination of SARS-CoV-2 cycle threshold will assist in decisions regarding infection control precautions.
